# Parent Perception of Child’s Behavior during the Initial Dental Visit among Children with Autism Spectrum Disorder: A Cross Sectional Study

**DOI:** 10.3390/ijerph20032454

**Published:** 2023-01-30

**Authors:** Marisa Chanin, Nicole Etcheverry, Maria A. Levi-Minzi, Jennifer Chung, Oscar Padilla, Romer A. Ocanto

**Affiliations:** 1 College of Dental Medicine, Nova Southeastern University, Fort Lauderdale, FL 33314, USA; 2 Abraham S. Fischler College of Education and School of Criminal Justice, Nova Southeastern University, Fort Lauderdale, FL 33314, USA

**Keywords:** autistic disorder, behavior, pediatric dentistry

## Abstract

(1) Background: The purpose of this study was to evaluate parent perception of behavior and level of cooperation to determine the success of a dental appointment with a child with autism spectrum disorder (ASD). (2) Methods: pre-treatment form, task analysis (TAS), and Frankl scale scores were extracted from patient charts. Values were calculated for patient demographics and other health characteristics (N = 235). Regression models were constructed to examine the success level during the first dental appointment (measured by TAS and Frankl scores) by several factors. (3) Results: The model to test patient characteristics: age, gender, ethnicity, and verbal communication, Hispanic ethnicity significantly predicted the TAS score, F (4, 191) = 2.45, *p* = 0.03 [95% CI −17.18, −3.53], and age significantly predicted the Frankl score, F (4, 194) = 5.17, *p* = 0.00 [95% CI 0.04, 0.12]. There was a significant association between parent perception of behavior and Frankl scores, F (2, 202) = 7.68, *p* = 0.00 [ 95% CI −0.11, −0.02]. (4) Conclusion: The results indicate that ethnicity and age play a role in successful outcomes during the dental appointment. Additionally, parent perception of their child’s behavior significantly predicted the Frankl score, thus coordinating with parents during the dental appointment can be a key factor in treatment planning for productive dental visits.

## 1. Introduction

About 1 in every 44 children are diagnosed with autism spectrum disorder (ASD), according to the Center for Disease Control [1]. ASD is prevalent in all racial, ethnic, and socioeconomic groups, and is four times more prevalent in males, compared to females. ASD is defined as a developmental disability that can cause significant impairments in social communications and interactions, and restricted and repetitive behaviors [1,2,3].

ASDs are neurobehavioral disorders often presenting within the first 2 years of life [4]. In the United States, pediatricians evaluate for ASD between 18 and 30 months of age, looking for possible signs including lack of eye contact, poor response to name called, or a significant regression in learned language skills [5,6]. Early intervention treatment is beneficial for patients that are diagnosed with ASD and can improve a child’s development [7]. Oral health care is one of the most prevalent unmet health care needs among U.S. children, especially those with ASD [3,8]. Children with ASD have a variable ability to cooperate and have a successful dental visit. There is a large range; some may be readily treated in the dental office, while others can have severe adverse reactions to the dental experience [9]. Additionally, studies in dentistry have concluded that children with ASD have a very high occurrence of certain comorbidities, such as developmental delay, intellectual disability and speech delay, which can further contribute as a barrier to their oral health care [10]. Patients with ASD have other unique barriers to receiving oral health care, including challenging behaviors, inadequate insurance benefits, and lack of trained or willing dentists [3,8]. The dental office environment poses a sensory overload for many children with ASD and parents may be hesitant to complete a dental visit using basic behavioral guidance techniques (BGTs) [11].

Collaborating with parents is a critical part in predicting the success of a dental visit for a child with ASD. Parents know the unique characteristics and needs of their child; therefore, parent perceptions should be taken into consideration when planning a patient’s individualized oral health care plan [12]. Although there are few studies that address parent perception regarding child behavior, one previous study found that parental accuracy in predicting cooperation of their autistic children varied by procedure, initial visits, and returning visits [13]. Gaining a more in-depth understanding of how parent perception of their child’s cooperation could be used as a useful tool for providers working in the dental setting.

The need for comprehensive oral health care in patients with ASD, coupled with the crucial role of parents in the success of the child’s dental appointments, provides the basis for this retrospective analysis. The purpose of this study is to assess parents’ ability to predict their child’s dental treatment cooperation during their first visit at the Mailman Segal Dental Clinic (MSDC), a pediatric special needs dental clinic tailored to patients aged 8 and under with ASD or other co-occurring special health care needs [3]. Two specific outcomes were assessed: the behavior of the child in the dental chair during their first appointment (as measured by the Frankl score) and the percentage of tasks the child was able to perform at that visit, or the productivity level of that appointment (as measured by the task analysis score (TAS). This study also aimed to examined the potential relationship between parental reported cooperation, behavior of their child, and the productivity level achieved. It was hypothesized that parents of children with ASD would be able to accurately predict their child’s behavior in the dental clinic at their first appointment. The results from this study can help dentists and health care professionals learn to use parents more effectively during appointments.

## 2. Materials and Methods

### 2.1. Cross Sectional Retrospective Chart Review

This project was approved by the Institutional Review Board at Nova Southeastern University (NSU IRB Protocol Number 2021-590). This was a retrospective chart review and therefore there was no patient contact; the collected data was de-identified in order to minimize risk and preserve patient rights. Given these procedures, a waiver of consent was approved for this study from the NSU IRB. Subjects were recruited from the MSDC, a pediatric special needs clinic serviced by pediatric dentistry residents and overseen by a pediatric dental specialist and a certified ABA specialist. This clinic specializes in monthly desensitization visits which helps patients to cope with dental treatment and facilitate the integration into their future dental home. A total of 476 patient charts were reviewed for children up to the age of 8 with an ASD diagnosis receiving dental services by residents and faculty at MSDC from 2012 through 2022. The inclusion criteria for this study consisted of the following: patients up to the age of 8, patients diagnosed with ASD, patients that are a part of the MSDC, and patients that have inactive or active status in axiUm. All charts that met the inclusion criteria (complete pretreatment form, Frankl score, and TAS score data) were included in this analysis (N = 235). All information was entered into a data entry form in REDCap. REDCap is a secure web application that captures data for clinical research and exports it into statistical programs and other data analysis software [14]. Patient chart numbers were used to identify each patient in REDCap to prevent any violation of patient privacy.

Parents of patients attending the clinic for the first time complete a pretreatment form aimed at collecting patient information, including demographics (age, sex, race, and ethnicity), past medical and dental history, social history, ASD diagnosis, at-home oral hygiene routine, and parent perception regarding their child’s ASD status, level of cooperation and challenging behaviors, and their preference in management techniques for the child in the dental office. For all subjects, the following information was collected from the axiUm electronic dental record: demographics, parent perceptions of their child’s anticipated behavior, the patient’s behavior during the visit, as measured by the Frankl score (a widely used tool in pediatric dentistry to evaluate the patient’s overall behavior) [15], and the level of cooperation during the visit, as measured by the task analysis score (TAS).

The Frankl scale, developed in 1962, is a tool that is widely used in pediatric dentistry to evaluate patient behavior. The Frankl score is a scale score used to measure patient behavior; it is calculated by the pediatric dentist during an appointment. The original scoring system categorized behavior into four groups: definitely negative, negative, positive, and definitely positive. In 1975, the following symbols were added to the categories: definitely negative (−−), negative (−), positive (+), and definitely positive (++), which made the scoring system even more popular. According to a 2017 study, the need for a further modification to the Frankl scale was made to include a fifth category: negative positive (−+) [15]. The Frankl scale is added to the provider’s note on axiUm. This scale will contribute to the overall evaluation of the behavior at the dental appointment.

At the MSDC, patients undergo desensitization treatment using task strips at every dental visit. The task analysis score (TAS) is a quantitative tool to measure the efficacy of desensitization during dental visits. It is calculated by the dental provider at the end of every visit, based on the number of completed tasks. This TAS value is compared from appointment to appointment to evaluate the progress of the patient’s success in desensitization. This score is based on the clinical task analysis form created and implemented as a method of evaluating the overall success of the dental appointment and the progress of desensitization. The task analysis form contains a total of 59 items of which three items range on a scale of time for acceptance of the item in the patient’s mouth. Following the completion of the task strip, the clinical task analysis form is automatically calculated based on the number of completed tasks and recorded into the axiUm patient record. The form in axiUm generates a percentage of completion (ranging from 0–100%) which will contribute to the overall evaluation of the success of the dental appointment.

### 2.2. Data Analysis

Descriptive statistics, including frequencies, means, and standard deviations were calculated for patient demographics and other health characteristics (N = 235). For the purpose of the analysis, the Frankl score was recorded to represent a range of 1–4, with higher values representing more cooperative behavior: [− −] definitely negative = 1; [−] negative = 2; [− +] some reluctance and some acceptance = 2.5; [+] positive = 3; and [+ +] definitely positive = 4. The TAS was scored as 0–100. The parental perception of the behavior score was calculated based on adding together scores from the following 3 questions with Likert Scale responses: “How would you describe your child’s level of challenging behavior?” (0 = none–3 = severe); “How often does your child engage in challenging behaviors?” (0 = never −3–3+ times daily), and “How would you describe your child’s ASD?” (0 = very minor–3 = severe). Parental perception of behavior scores ranged from 0–9, with higher scores representing more perceived challenging behaviors. Regression models were constructed to examine the productivity level achieved during the first dental appointment (measured by outcome variables TAS and Frankl scores) by the following factors: demographics (patient age, gender, ethnicity, verbal communication), parental perception (behavior score and level of challenging behaviors), past dental visit and patient dental needs, and selected caregiver behavioral management techniques. The regression analysis included an N of 205 due to missing data on some of the pertinent variables. A value of *p* ≤ 0.05 was significant.

Linearity was assessed by partial regression plots and a plot of studentized residuals against the predicted values. Visual inspection of the plots of studentized residuals versus unstandardized predicted values found independence of residuals and homoscedasticity. There was no evidence of multicollinearity, as assessed by tolerance values greater than 0.1. There were no studentized deleted residuals greater than ±3 standard deviations, no leverage values greater than 0.2, and values for Cook’s distance above 1. The assumption of normality was met, as assessed by Q-Q Plots.

## 3. Results

Patient demographics were collected for the sample (N = 235). As indicated in Table 1, the majority of patients were male (82.1%), and of Caucasian race (44.7%), followed by multi-race (9.8%), African American (9.4%), Asian American (3.4%), and American Indian (0.4%); 32.3% declined to report race. Hispanic ethnicity was reported by 29.4% of the sample.

Patients’ health characteristics were also collected (Table 2). Most of the patients (N = 92.8%) already had an ASD diagnosis prior to their first dental visit at the MSDC, and of those reporting, 27.2% described a moderate level of ASD. The most commonly co-occurring disorders were speech delay (39.1%) and developmental delay (24.3%). Parents of about 30 children reported their child taking prescribed medications. The majority of patients were receiving other professional services which mainly included speech therapy (56.6%), occupational therapy (46.0%), applied behavior analysis (35.3%), and physical therapy (6.8%). Of those that reported patient communication style, 36.6% used nonverbal communication. Regarding at home dental care, the majority of participants used a manual toothbrush (48.5%) over an electric toothbrush (15.7%), and the use of fluoride toothpaste and dental floss were low. In terms of dental treatment history (Table 3), 38.7% reported a prior dental visit. When asked about their child’s dental needs, the majority of parents responded with routine exam (83.0%) or cleaning (56.6%), while some parents were unsure (16.6%).

In terms of behaviors (Table 4), the majority of parents were unsure whether their child would cooperate (30.6%). When asked about the best management technique to use during the dental appointment, the majority were unsure (54.0%), 36.2% thought their child would require short, multiple visits, 15.3% believed the child would need sedation, followed by 8.9% restraint, and 3.0% general anesthesia. Regarding the parents’ rating of challenging behavior, the majority perceived moderate levels of challenging behavior (29.4%). In terms of frequency of challenging behaviors, 26.8% reported 1–2 per day.

The mean TAS for the sample was 81.34 (SD = 19.84; range = 13–100), suggesting that children attending the clinic for the first time were cooperative and able to complete several of the attempted tasks. The mean Frankl score for the sample was 2.81 (SD = 0.85; range = 1–4). These results indicate that patients had moderate levels of cooperative behavior. The mean score on the parental perception scale was 3.35 (SD = 2.81; range = 0–9), with higher scores representing more challenging behaviors; given this, it seems the children seen in our clinic exhibit low levels of challenging behaviors.

**Regression results: TAS Scores (Table 5).** The model to test patient characteristics, including age, gender, race, ethnicity, and verbal communication, Hispanic ethnicity significantly predicted TAS, *F* (4, 191) = 2.45, *p* = 0.03 [95% CI −17.18, −3.53]. Hispanic patients had a significantly lower TAS score than non-Hispanic patients. The model to test the association between parent perception of behavior and TAS was not significant, and patient dental visit characteristics, including treatment needs and dental history did not significantly predict TAS, *F* (3, 197) = 0.07, [95% CI 74.08, 89.38]. Behavioral management techniques predicted by the caregiver also did not significantly predict TAS, *F* (3, 198) = 1.16, [95% CI 74.80, 87.36].

**Regression results: Frankl Scale (Table 6).** In the model to test patient characteristics, including age, gender, ethnicity, and verbal communication, age significantly predicted the Frankl score, *F* (4, 194) = 5.17, *p = 0.00* [95% CI 0.04, 0.12]; older patients had significantly higher Frankl scores (i.e., more cooperative behavior). The model to test the association between parent perception of behavior and Frankl scores was also significant, *F* (2, 202) = 7.68, *p = 0.00* [ 95% CI −0.11, −0.02]. An increase in parent perception of challenging behaviors was associated with a decrease in the Frankl score; as challenging behaviors increased, Frankl scores (i.e., cooperative behavior) significantly decreased.

Patient dental visit characteristics, including treatment needs and dental history did not significantly predict the Frankl score *F* (3, 200) = 1.47, [95% CI 2.23, 2.86], and behavioral management techniques suggested by the parent did not significantly predict the Frankl score, *F* (3, 201) = 0.20, [95% CI 2.54, 3.05].

## 4. Discussion

This cross-sectional study examined patient demographics, health characteristics, and parent perception in relation to the level of success achieved at a dental visit among children with ASD at a special needs clinic. Upon reviewing patient demographics from this population, it was noted that there was a range of co-morbidities, including developmental delay, intellectual delay, and speech delay. In our study, it was noted that children with ASD may have different varying levels of challenging behaviors and cooperation. Challenging behaviors can include the following: non-verbal or minimal use of language, inability to understand language at an age-appropriate level, and the inability to follow instructions [13]. These behaviors were reflected in the chosen Frankl score for each patient, indicated by a wide range of Frankl scores.

This study revealed that there is a difference between behavior and cooperation; therefore, these terms should not be used interchangeably. Patients can exhibit high levels of cooperative behavior (Frankl) but have sensitivities when it comes to their dental experience (i.e., audiovisual stimuli, tastes, and oral stimuli), so they may not be as cooperative (TAS) when it comes to tasks that could trigger their particular sensitivities [16]. A possible result of this dichotomy is that cooperation (TAS) could be affected more significantly than behavior (Frankl).

Although parent prediction of their child’s behavior did not significantly predict the TAS score, it did significantly predict the Frankl score; therefore, the parent was able to accurately predict their child’s behavior, but was not able to predict their cooperation level for particular tasks presented at the initial dental visit. A reason for this could be that the Frankl score is more sensitive and subjective than the TAS. As the mean score increased, the parental perception scale for challenging behavior decreased, which meant that parents were able to accurately predict their child’s challenging behavior.

It was also found that age was significantly correlated with the Frankl score. In a previous study that used the Frankl scale as its behavioral assessment, it was noted that age, by itself, was the best predictor for an association with disruptive behavior [17]. In this study, it was found that older patients had significantly higher Frankl scores (i.e., better overall behavior). This suggests that the Frankl score is a more accurate measure than the TAS, even though there is some subjectivity.

The findings also indicate that ethnicity predicted the TAS. It was found that children with Hispanic ethnicity had a significantly lower TAS than their non-Hispanic counterparts. One factor that could contribute to patient cooperation for completing tasks is language. Language has been identified as a central impeding factor in health care treatment for U.S. Hispanic families that care for children with ASD [18]. This could be due to the fact that not all providers are Spanish-speaking, and there may have been a lack of proper communication with the patient at their initial visit. Although the provider may not always be Spanish speaking, the assistants, faculty, or translation is accessible to ensure proper communication. Language and cultural barriers can also account for lower TAS scores among Hispanic children. Additionally, they may experience barriers when seeking out needed care, including but not limited to, cultural and linguistic factors, unfamiliarity, fear, and mistrust of health care systems [19].

The type of procedure, prior dental visits, and parents’ anticipated management techniques did not have an impact or an association with the TAS or Frankl score. Parents’ perception of what dental procedures the patient needed was therefore not predictive of the behavior or cooperation at the first visit.

A limitation of this study is that participants were from the MSDC in Fort Lauderdale, FL. The results of this study may not be comprehensive of patients with special health care needs in other geographical locations. Other limitations include the subjective nature of the Frankl scale, the variation in the task analysis score, and the information collected from pre-treatment assessment forms.

Evaluating behavior cannot easily be specified, standardized, or objectified, which causes inaccuracy in the reliability, validity, and measurement levels of the results [20]. There is no definitive analytical tool to identify patient behavior; therefore, variations of the Frankl scale were seen in the charts due to different providers who saw the patients on different dates. The drawback of this is that there is possible undetected bias and misrepresentation of data [20]. The Frankl rating scale does not effectively communicate a patient’s range of behaviors, especially when using the symbols, + or −, unless otherwise noted in the patient’s chart. Many charts reviewed had a behavioral description associated with their Frankl symbol. In this study, we did not evaluate the written description of behavior, due to the subjectivity of the description. While this may be non-specific or nondiagnostic, it can still help the provider for future appointments, based on their past behaviors [21].

Another limitation was found when reviewing the TAS in patient charts. The percentage generated from the TAS form reflects the tasks that were offered and completed; however, was not calculated from the entirety of the tasks on the form. In addition, different providers completed the TAS forms. Thus, it was difficult to find a true correlation based on the TAS calculated score alone since the percentage does not indicate how many tasks were completed.

## 5. Conclusions

These findings illustrate the importance of the parents’ role in assisting the dental team. Particularly among children with ASD, parents may be a useful asset in determining the outcome of a dental appointment since they likely know best what their child needs. However, while a parent may be able to accurately predict their child’s behavior, they may not be able to accurately predict their cooperation level that is needed in a dental setting.

Our results can help provide pediatric dentists and other health care professionals tools to better assist parents with assessing their child’s anticipated behavior and cooperativity level during the initial visit. This can benefit the patient by bringing techniques learned at the initial visit home to enhance daily oral health care routines and prepare for future successful dental appointments.

## Figures and Tables

**Table 1:** Patient demographics.

Mean Patient Age: 7.95 (SD = 2.76, Range = 3–14)		
Variable	N	%
**Gender** ^1^		
Male	193	82.10%
Female	41	17.40%
**Race**		
Caucasian	105	44.70%
Multi-Race	23	9.80%
African American	22	9.40%
Asian	8	3.40%
American Indian	1	0.40%
Unknown/Not Reported	76	32.30%
**Hispanic Ethnicity**		
Hispanic	69	29.40%
Non-Hispanic	48	20.40%
Unreported	118	50.20%

^1^ Data missing for 1 participant.

**Table 2:** Patient health characteristics.

	N	%
ASD Diagnosis	218	92.80%
**ASD Level**		
Mild	55	23.40%
Moderate	64	27.20%
Severe	10	4.30%
Other	76	32.30%
**Co-Occurring Disorders**		
Speech Delay	92	39.10%
Developmental Delay	57	24.30%
Prescribed Medication	30	12.80%
**Other Services Patient is Receiving**		
Speech Therapy	133	56.60%
Occupational Therapy	108	46.00%
ABA *	83	35.30%
Physical Therapy	16	6.80%
**Patient Communication Style**		
Uses Nonverbal Communication	86	36.60%
Can Communicate Verbally	83	35.30%
**At Home Dental Care**		
Manual Toothbrush	121	48.50%
Electric Toothbrush	37	15.70%
Toothpaste with Fluoride	84	35.70%
Uses Floss	31	13.20%

* ABA = Applied Behavior Analysis.

**Table 3:** Patient dental visit characteristics.

	N	%
**Patient Dental Needs**		
Routine Exam	195	83.00%
Cleaning	133	56.60%
Not Sure	39	16.60%
Fillings	13	5.50%

**Table 4:** Patient behavioral characteristics.

	N	%
**Caregiver Perceived Level of Patient Cooperation**		
Short Attention Span	65	27.70%
Not Sure	72	30.60%
Non-Focused	51	21.70%
Age Appropriate	49	20.90%
Aggressive	46	19.60%
Playful	34	14.50%
**Caregiver Perceived Best Management Technique to Use During Appointment**		
Not Sure	127	54.00%
Short Multiple Visits	85	36.20%
Sedation	36	15.30%
Restraint	21	8.90%
OR/General Anesthesia	7	3.00%
**Caregiver Rating of Challenging Behavior**		
**Level of Challenging Behavior**		
Minimal	52	22.10%
Disruptive (moderate)	69	29.40%
Severe (high)	16	6.80%
Not Applicable	98	41.70%
**Frequency of Challenging Behaviors**		
<1 per day	32	13.60%
1–2 per day	63	26.80%
3+ per day	38	16.20%
Not Applicable	100	42.60%

**Table 5:** Regression analysis to predict productivity level achieved during the first dental appointment, as measured by TAS Score (N = 205).

	95% CI for *B*		*p* Value	Δ *R*^2^
	*LL*	*UL*		
**Patient Characteristics**				
Model				0.03
Constant	70.16	94.16		
Child Age	−1.15	1.00	0.88	
*Child Race/Ethnicity Hispanic*	*−17.18*	*−3.53*	*0.03*	
Male Gender	−7.30	7.34	0.99	
Verbal Communication	−2.38	10.31	0.22	
**Caregiver Perception Behavior**				
Model				−0.08
Constant	76.83	87.49		
Parent perception of behavior score	−1.41	0.77	0.56	
Parents reported 1–2 challenging behaviors a day	−6.72	5.43	0.83	
**Patient Dental Visit Characteristics**				
Model				−0.04
Constant	74.08	89.38		
Routine Exam	−8.89	7.06		
Cleaning	−6.11	5.95		
Has visited the dentist before	−3.66	2.46		
**Behavioral Management Techniques**				
Model				0.00
Constant	74.80	87.36		
Not Sure	−4.54	8.86	0.53	
Multiple Visits	−10.17	3.40	0.33	
Sedation	−10.25	5.88	0.59	

Note: Model = “Enter” method in SPSS; B = unstandardized regression coefficient; CI = confidence interval; *LL* = lower limit; *UL* = upper limit; Δ *R*^2^ = adjusted *R*^2^.

**Table 6:** Regression analysis to predict productivity level achieved during the first dental appointment, as measured by Frankl (N = 205).

	95% CI for *B*		*p* Value	Δ *R*^2^
	*LL*	*UL*		
**Patient Characteristics**				
Model				0.08
Constant	1.77	2.71		
*Child Age*	*0.04*	*0.12*	*0.00*	
Child Race/Ethnicity Hispanic	−0.47	0.07	0.14	
Male Gender	−0.39	0.19	0.49	
Verbal Communication	−0.02	0.47	0.08	
**Caregiver Perception Behavior**				
Model				0.06
Constant	2.94	3.37		
*Parent perception of behavior score*	*−0.11*	*−0.02*	*0.00*	
Parents reported 1–2 challenging behaviors a day	−0.39	0.90	0.22	
**Patient Dental Visit Characteristics**				
Model				0.00
Constant	2.23	2.86		
Routine Exam	−0.17	0.49	0.37	
Cleaning	−0.15	0.34	0.45	
Has visited the dentist before	−0.03	0.22	0.15	
**Behavioral Management Techniques**				
Model				−0.01
Constant	2.54	3.05		
Not Sure	−0.25	0.30	0.85	
Multiple Visits	−0.26	0.30	0.88	
Sedation	−0.44	0.21	0.50	

Note: Model = “Enter” method in SPSS; B = unstandardized regression coefficient; CI = confidence interval; *LL* = lower limit; *UL* = upper limit; Δ *R*^2^ = adjusted *R*^2^.

## Data Availability

Not applicable.
